# Renal mucinous cystadenoma in the context of lynch syndrome and colonic neuroendocrine neoplasm: a case report

**DOI:** 10.1093/omcr/omaf012

**Published:** 2025-03-28

**Authors:** Ivy Wen, Lawrence H Kim, Matt Wong, Shannon di Lernia, Fiona Maclean, Allen Lee, Adrian Lee, Anu Ganapathy, David Chan

**Affiliations:** Department of Medical Oncology, Royal North Shore Hospital, Reserve Road, St Leonards, 2065, Sydney, New South Wales, Australia; Department of Urology, Westmead Hospital, Hawkesbury Road, Westmead, 2145, Sydney, New South Wales, Australia; University of Sydney, Camperdown, 2050, Sydney, New South Wales, Australia; University of Newcastle, University Drive, Callaghan, 2308, New South Wales, Australia; Department of General Surgery, Gosford Private Hospital, Burrabil Avenue, Gosford, 2250, New South Wales, Australia; Anatomical Pathology, Douglass Hanly Moir Pathology, Giffnock Avenue, Macquarie Park, 2113, Sydney New South Wales, Australia; Department of Nuclear Medicine, Royal North Shore Hospital, Reserve Road, St Leonards, 2065, Sydney, New South Wales, Australia; Department of Medical Oncology, Royal North Shore Hospital, Reserve Road, St Leonards, 2065, Sydney, New South Wales, Australia; University of Sydney, Camperdown, 2050, Sydney, New South Wales, Australia; Department of Medical Oncology, Royal North Shore Hospital, Reserve Road, St Leonards, 2065, Sydney, New South Wales, Australia; Department of Medical Oncology, Royal North Shore Hospital, Reserve Road, St Leonards, 2065, Sydney, New South Wales, Australia; University of Sydney, Camperdown, 2050, Sydney, New South Wales, Australia

**Keywords:** renal mucinous cystadenoma, neuroendocrine carcinoma, lynch syndrome, MiNEN

## Abstract

Renal mucinous cystadenoma (RMC) is an exceptionally rare finding with a poorly understood pathogenesis. Links between RMC and other malignancies are not well described, nor are there known associations with familial cancer disorders. We present the first case of RMC associated with Lynch syndrome (LS) and neuroendocrine neoplasm. A 56-year-old woman presented with iron deficiency leading to a diagnosis of a colonic mixed neuroendocrine-non-neuroendocrine carcinoma. After initial surgery, she experienced local recurrences at 6 and 12 months, treated with resection and adjuvant chemotherapy. Tumours displayed deficient MMR proteins with *BRAF* positivity, and germline testing confirmed LS. Surveillance subsequently revealed a complex cyst arising from a horseshoe kidney, for which she underwent a partial nephrectomy. Histopathology confirmed this to be renal mucinous cystadenoma arising from the pelvicalyceal system. This case underscores the need for further investigation into RMC pathogenesis and its potential association with LS.

## Introduction

Renal mucinous cystadenoma (RMC) is an extremely rare, typically benign tumour with potential for malignant transformation. Fewer than 30 cases have been described worldwide—due to its rarity, associations with genetic syndromes and links to other malignancies are not well-documented [1]. To date, no reports exist of RMC in the context of a neuroendocrine neoplasm (NEN) nor a known familial cancer disorder. Lynch syndrome (LS) is an autosomal dominant hereditary cancer syndrome caused by germline mutations in mismatch repair (MMR) genes, with prevalence of 3% of colorectal cancers [2]. Less well-described is occurrence specifically with colonic neuroendocrine neoplasms. NENs, encompassing both well-differentiated tumours and poorly-differentiated carcinomas are uncommon but increasing in incidence [3]. We present a case report of a lady with metachronous renal mucinous cystadenoma and colonic mixed neuroendocrine-non-neuroendocrine carcinoma in the setting of LS.

## Case report

A 56-year-old woman presented to clinic with iron deficiency. Her medical history included hypertension and non-alcoholic steatohepatitis. Family history of malignancy was unremarkable, with limited paternal history due to adoption.

A computed tomography (CT) chest/abdomen/pelvis scan demonstrated a transverse colon lesion, confirmed on biopsy as large cell adenocarcinoma with neuroendocrine differentiation. The patient underwent extended right hemicolectomy. Histology confirmed completely resected stage IIA (pT3pN0) moderately differentiated adenocarcinoma with a 10% component of neuroendocrine carcinoma, on the basis of positive staining for synaptophysin and chromogranin—not present in the adenocarcinoma components. The Ki-67 index was near 100%. Immunohistochemistry (IHC) showed loss of *PMS2* staining, with *BRAF* positivity.

**Figure 1:**
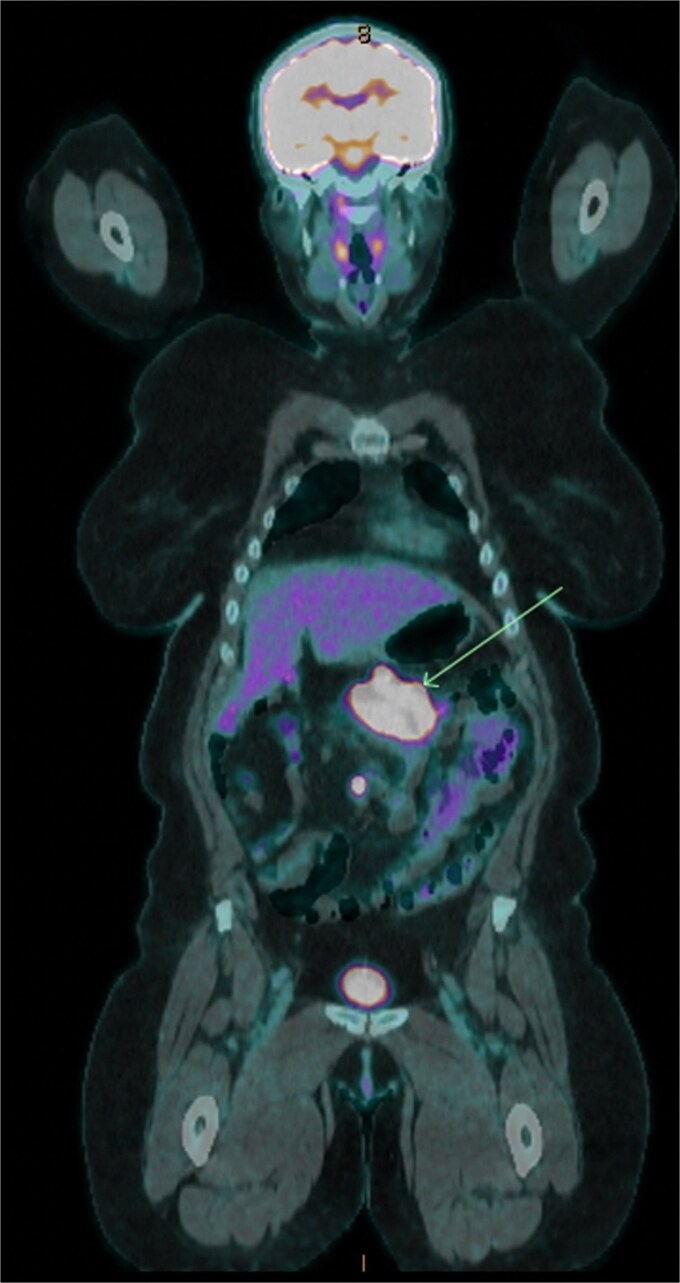
18F-FDG positron emission tomography/computed tomography showing transverse colonic recurrence (arrow).

Six months following surgery, she developed anastomotic recurrence, without distant metastases on restaging (Fig. 1). She underwent ileocaecal resection, histology confirming a pure neuroendocrine carcinoma (NEC) (Ki-67 90%), with nodal involvement and clear margins. IHC again showed loss of *PMS2* with *BRAF* positivity. She completed three months of adjuvant capecitabine & oxaliplatin.

Colonoscopy six months post-operatively revealed a second anastomotic recurrence. On further ileocolic resection, pathology again revealed NEC. The patient opted for active surveillance. Germline testing revealed a *PMS2* likely pathogenic variant, confirming LS.

**Figure 2:**
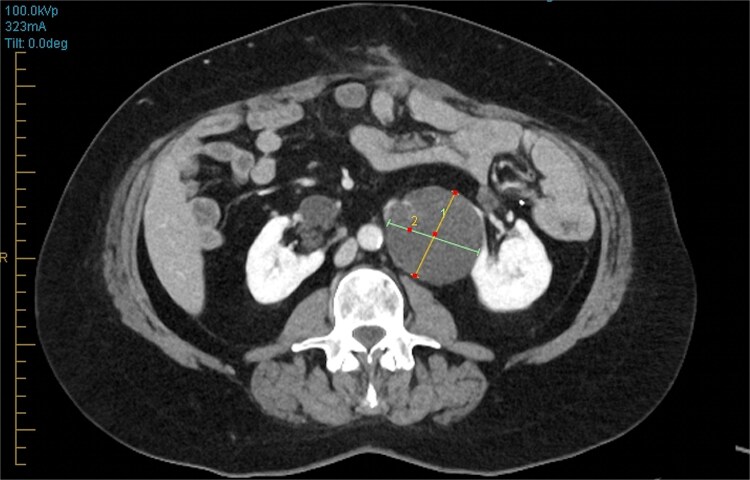
CT abdomen/pelvis axial slice showing complex left sided renal cyst.

Three years into surveillance, enlargement of a previously stable left renal cyst (57x55mm, previously 45x41mm), arising from a horseshoe kidney was noted. CT showed a complex bilobed cystic lesion with irregular enhancing septae, consistent with a Bosniak III lesion (Fig. 2).

**Figure 3:**
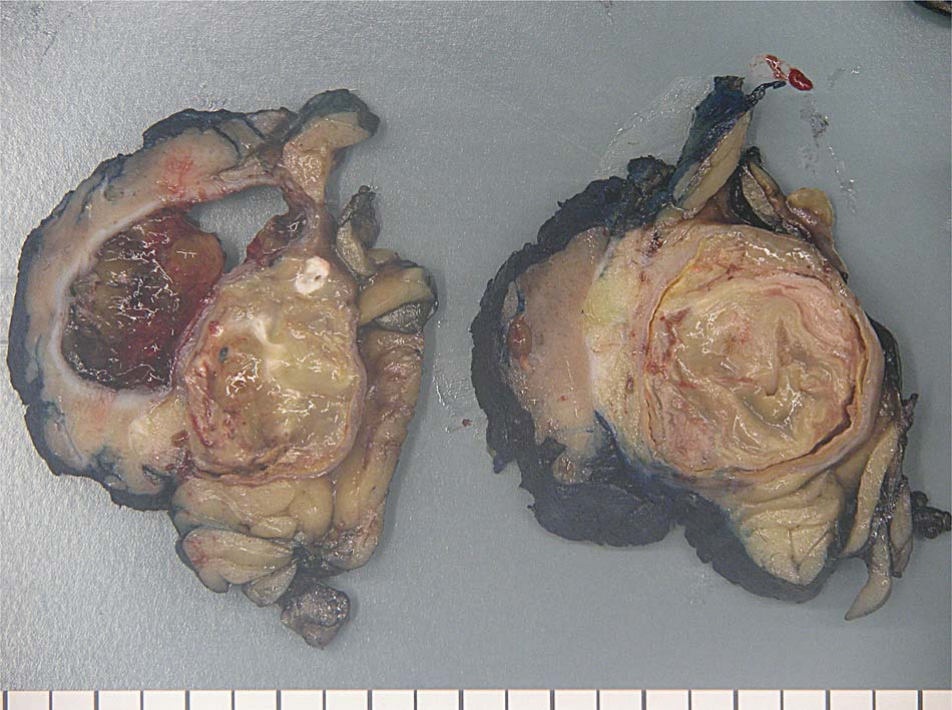
Renal mucinous cystadenoma pathology.

**Figure 4:**
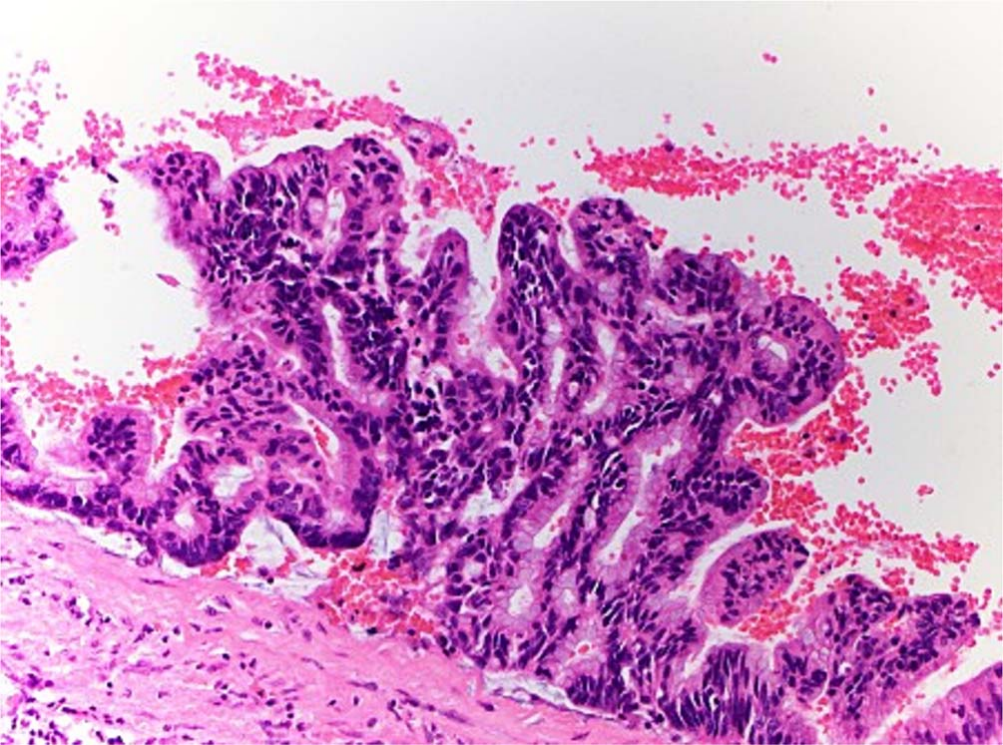
Microscopic pathology of renal mucinous cystadenoma.

The patient therefore proceeded directly to left robotic partial nephrectomy. The tumour measured 45x30x25mm and contained green/brown mucinous material (Fig. 3). Microscopically, the cystic neoplasm was lined by mucinous epithelium and surrounded by dense fibrovascular tissue (Fig. 4). A single area of attenuated urothelium merged with the cystadenoma epithelium, indicating connection with the pelvicalyceal system. *BRAF* IHC was negative, with preserved nuclear staining for MMR proteins. The final diagnosis was RMC arising from the pelvicalyceal system.

The patient returned to active surveillance, without recurrence of RMC or NEC 12 months post-resection.

## Discussion

Renal mucinous cystadenoma is exceedingly rare, with only 23 cases reported. These tumours can arise from the pelvicalyceal system or parenchyma. Those associated with the renal pelvis are usually in the context of chronic irritation secondary to prior stones or pyelonephritis [1]. Few case reports describe occurrence in association with maldevelopment (as with horseshoe kidneys), potentially arising from a sequestered segment of renal pelvic epithelium within the parenchyma [4]. This is the fourth reported case of RMC in a horseshoe kidney, and the first associated with LS [4–6]. Further investigation is needed to understand RMC pathogenesis, and potential association with LS pathways remain unknown. Interestingly our patient’s RMC demonstrated proficient MMR status, an unusual finding in the setting of LS.

Diagnosis of RMC is often made only following excision, as they can be radiologically indistinguishable from renal cysts. Histologically, RMC features mucin-producing columnar epithelium lining the inner surface. Those arising from the pelvicalyceal system can be differentiated by their transition to urothelium, as in this case [1]. Complete surgical excision is recommended due to the risk of malignant transformation. Due to its rarity, no established post-resection surveillance recommendations exist. Whilst case reports have generally not reported any local or metastatic recurrence, more published data will confirm excellent post-resection outcomes.

NENs encompass well-differentiated neuroendocrine tumours, with a typically indolent biology, and the poorly-differentiated, aggressive neuroendocrine carcinomas [3]. Mixed tumours with both neuroendocrine and non-neuroendocrine components are known as MiNENs if each component exceeds 30% of the total tumor. However, presence of even 10%–30% neuroendocrine differentiation portends a significantly poorer prognosis than adenocarcinoma alone [7]. Amongst this heterogeneous group, use of dual PETs have been proposed as a prognostic biomarker, with FDG-positive/DOTATE-negative NENs associated with significantly lower overall survival [8].

Emerging evidence suggests a potential association between NEC and LS. While LS’s association with adenocarcinomas is well-established, reports have also described NEC in LS, with tumours exhibiting loss of MMR protein expression, and high microsatellite instability, a hallmark of LS [9]. In our patient, both the initial and subsequent NECs demonstrated loss of *PMS2* staining, consistent with LS. The association with the RMC, which demonstrated proficient MMR status, is less clear. Beyond the occurrence of two rare tumour types, other unusual elements of this case include the pattern of multiple local recurrences without metastatic disease, and a relatively long period of relapse free survival- unusual for NECs which have a propensity for rapid progression and early metastasis. Additionally, microsatellite instability with confirmed Lynch syndrome is rarely associated with *BRAF* positivity.

Whether the susceptibility of MSI-H NECs to immunotherapy resembles that of MSI-H CRCs is yet to be investigated in a prospective trial, but is a reasonable research avenue given around 12% of gastroenteric NECs are MSI-H [10]. This is particularly pertinent given the FDA’s tumour-agnostic approval of pembrolizumab for MSI-H cancers. Furthermore, should there be additional cases of RMC synchronous with other tumours, genomic sequencing could provide insights into the genetic foundations of RMC formation and potential links to other tumours.

## Conclusion

Renal mucinous cystadenoma is an extremely rare tumour for which underlying pathogenesis requires further investigation. This is the first reported case of RMC in association with a familial cancer disorder and neuroendocrine carcinoma.

## Consent

This case study has been published with the consent of the patient involved.

## Guarantor

Dr Ivy Wen.
